# Regional and developmental brain expression patterns of SNAP25 splice variants

**DOI:** 10.1186/1471-2202-12-35

**Published:** 2011-04-28

**Authors:** Gerald R Prescott, Luke H Chamberlain

**Affiliations:** 1 Centre for Integrative Physiology, School of Biomedical Sciences, Hugh Robson Building, University of Edinburgh, Edinburgh EH8 9XD, UK

## Abstract

**Background:**

SNAP25 is an essential SNARE protein for regulated exocytosis in neuronal cells. Differential splicing of the SNAP25 gene results in the expression of two transcripts, SNAP25a and SNAP25b. These splice variants differ by only 9 amino acids, and studies of their expression to date have been limited to analysis of the corresponding mRNAs. Although these studies have been highly informative, it is possible that factors such as differential turnover of the SNAP25 proteins could complicate interpretations based entirely on mRNA expression profiles.

**Results:**

We report the generation and characterization of antibodies that distinguish between SNAP25a and SNAP25b isoforms, and their use to investigate the expression profile of these proteins in rat and human brain. In rat brain, SNAP25b protein expression increased dramatically during post-natal development, whereas the increase in SNAP25a expression was more modest and variable. The extent of this up-regulation in SNAP25b expression was similar across cortex, cerebellum and hippocampus. The SNAP25 isoforms also displayed distinct regional expression patterns, with SNAP25a very weakly expressed in both rat and human cerebellum. Quantitative analysis revealed that SNAP25b was the dominant isoform in all adult human brain regions examined.

**Conclusions:**

SNAP25a and SNAP25b display distinct developmental and regional expression profiles in rat and human brain. These differences might reflect distinct functions of these highly conserved isoforms in membrane fusion pathways in the brain. The antibodies generated and characterized in this study represent important tools for future analyses of these essential SNARE protein isoforms.

## Background

Exocytosis, the fusion of intracellular secretory vesicles with the plasma membrane, is essential for protein targeting and for secretion of soluble vesicle components to the extracellular milieu. This pathway occurs constitutively in all cell types but can also be a highly regulated process, such as synaptic vesicle exocytosis in neurons. The exocytosis of synaptic vesicles is driven by interactions between the plasma membrane SNARE proteins syntaxin 1 and SNAP25 and the vesicle SNARE VAMP2 [1-3]. These neuronal SNARE proteins are specific targets of the potent botulinum and tetanus neurotoxins, emphasizing their essential functions in synaptic vesicle fusion events [4-9]. In contrast to syntaxin 1 and synaptobrevin, which are transmembrane proteins, SNAP25 is synthesized as a soluble protein and is anchored to membranes via the palmitoylation of a central cysteine-rich cluster [10,11].

SNAP25 is expressed as alternatively spliced isoforms, SNAP25a and SNAP25b [12,13]. These splice variants differ by only 9 out of 206 amino acids, a result of differential usage of two alternative exon 5 sequences (exon 5a/5b). Interestingly, three of the non-conserved residues occur within the cysteine-rich domain, altering the configuration of the palmitoylated cysteines. These differences in the cysteine-rich domains of SNAP25a and SNAP25b may affect their interaction with palmitoyl transferases [14], and the precise intracellular targeting of the proteins [15,16].

Whereas SNAP25a/b expression is restricted to neuronal cells and a small number of cells outside the central nervous system (adrenal medullary chromaffin cells and pancreatic beta cells), SNAP23 is expressed ubiquitously [17,18]. SNAP23 is ~ 60% identical to SNAP25, and has been proposed to function in both regulated exocytosis and constitutive membrane fusion events [19-21].

Elegant electrophysiological studies using adrenal medullary chromaffin cells from SNAP25 null mouse embryos revealed that over-expression of either SNAP25a or SNAP25b rescues exocytosis [22]. Interestingly though, SNAP25b supports more exocytosis than SNAP25a in this system, clearly showing that the isoforms do not have directly interchangeable functions [22]. Indeed, transgenic mice in which exon 5b is replaced with an extra copy of exon 5a (leading to the exclusive expression of the SNAP25a isoform) exhibit developmental defects, seizures and impairment of learning [23].

Despite the importance of the SNAP25 splice variants, there have been no comparative analyses of endogenous SNAP25a and SNAP25b protein expression; this is entirely due to a lack of suitable antibodies to distinguish between the splice variants. As a result, all characterization of endogenously expressed SNAP25a/b has been performed at the mRNA level. Interestingly, in mice, SNAP25a and SNAP25b transcript levels are broadly similar until around two weeks after birth, following which there is a dramatic up-regulation of SNAP25b levels [15]. In contrast, levels of SNAP25a mRNA exhibit at best a marginal increase during post-natal development in the same samples [15]. A similar post-natal increase in SNAP25b expression was reported for rat brain [24]. The mRNA encoding SNAP25a and SNAP25b also exhibit differences in regional expression, for example, at P14 SNAP25a was enriched in layer IV of the cortex, whereas SNAP25b was more abundant in the outer and inner cortical layers [15]. Whereas SNAP25b mRNA is the most abundant isoform expressed in many post-natal brain regions, SNAP25a mRNA is the major isoform in adrenal and pituitary glands and in neuroendocrine PC12 cells [15,25].

Analysis of the mRNA expression profiles of SNAP25a and SNAP25b has provided important information on the relative expression of these SNAP25 splice variants. However there is not always a direct correlation between mRNA levels and protein expression [23]. Here we describe the generation and characterization of antibodies that can distinguish between SNAP25a and SNAP25b, and their use to investigate developmental and regional patterns of expression of the splice variants in rat and human brain.

## Results

### Generation and characterization of antibodies that selectively recognize either SNAP25a or SNAP25b

**Figure 1:**
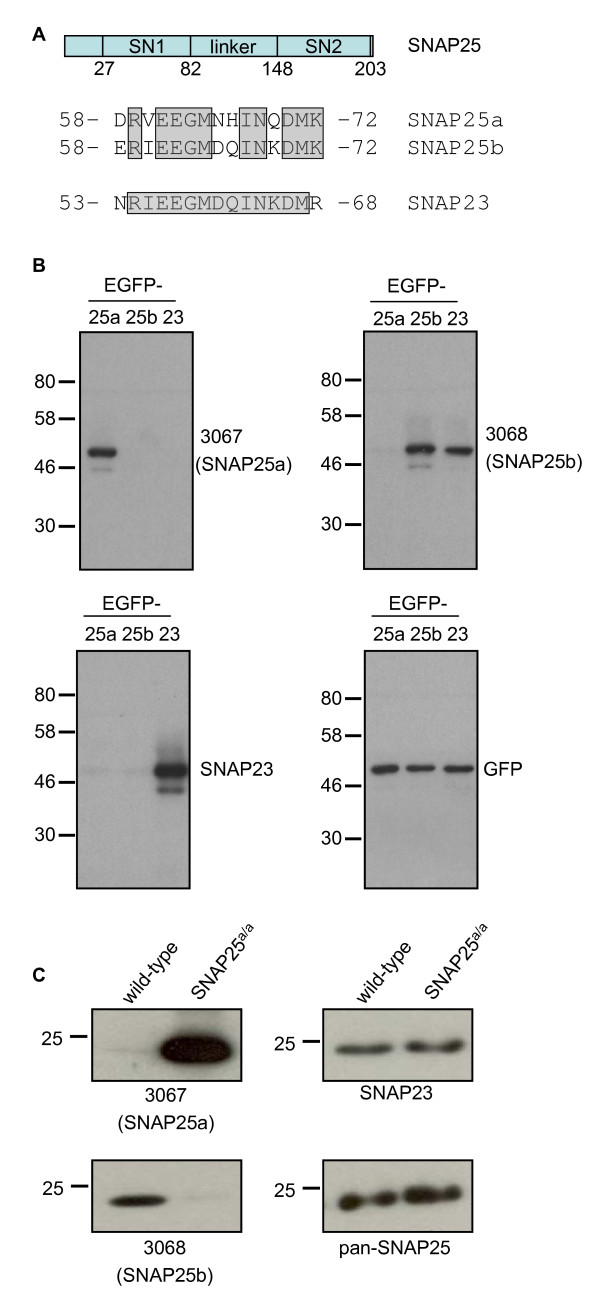
**Characterization of 3067 and 3068 antibodies**. A) The top schematic (in blue) shows the domain structure of SNAP25; SN1 and SN2 denote the respective SNARE domains. An alignment of the peptide sequences from rat SNAP25a and SNAP25b that were used for antibody production is also shown. The sequence of rat SNAP23 in the corresponding region is provided for comparison. Numbers indicate the position of the peptides in relation to the full-length proteins. The shading highlights conserved residues in SNAP25a and SNAP25b. The shading for SNAP23 highlights amino acids that are identical to those in the corresponding region of SNAP25b. B) HEK293T cells were transfected with plasmids encoding EGFP-tagged SNAP25a, SNAP25b and SNAP23. Equal volumes of lysates from the transfected cells were probed with antibodies against SNAP25a (3067), SNAP25b (3068), SNAP23 (Synaptic Systems), and GFP (Roche). C) Cerebellum lysates from wild-type or SNAP25^a/a ^mice were resolved by SDS-PAGE and transferred to nitrocellulose for immunoblotting analysis using 3067, 3068, pan-SNAP25 and SNAP23 antibodies. The position of molecular weight markers are shown on the left side of panels B and C.

To study the expression profile of SNAP25a and SNAP25b proteins, rabbits were immunized with peptides that correspond to non-conserved regions of the isoforms present within the differentially spliced exon 5 (Figure 1A). These different peptides did not contain more than four identical contiguous amino acids and only 10 out of 15 residues were identical (Figure 1A). IgG fractions were purified from the collected antisera: IgG #3067 was from rabbits immunized with the SNAP25a peptide and IgG #3068 was from rabbits immunized with the SNAP25b peptide.

To test the specificity of the antibodies, lysates were prepared from HEK293T cells that had been transfected with EGFP-SNAP25a, EGFP-SNAP25b or EGFP-SNAP23. IgG 3067 specifically recognized SNAP25a, and IgG 3068 detected SNAP25b but not SNAP25a (Figure 1B). Figure 1A shows that the sequence of SNAP23 in the region used for peptide synthesis is very similar to SNAP25b; consistent with this, the 3068 antibody also displayed immunoreactivity against SNAP23 (Figure 1B).

To examine whether the 3067 and 3068 antibodies could distinguish between endogenously expressed SNAP25a and SNAP25b, lysates were prepared from cerebellum samples from wild-type mice and from mice in which exon 5b is replaced with an additional copy of exon 5a (SNAP25^a/a^) [23]. As a result of this replacement, these transgenic mice exclusively express SNAP25a. The 3067 and 3068 antibodies exhibited very high selectivity towards endogenous SNAP25a and SNAP25b, respectively (Figure 1C). Total SNAP25 and SNAP23 levels were not noticeably different between the samples (Figure 1C), and the massive increase in SNAP25a levels in the SNAP25^a/a ^cerebellum reflects the much higher expression of the SNAP25b isoform in this brain region of wild-type animals (see later). The very faint residual band detected by the 3068 antibody in the cerebellum of SNAP25^a/a ^mice is presumably SNAP23, highlighting the vast excess of SNAP25b protein compared with SNAP23 in brain samples. This observation is important as it shows that cross-reactivity with SNAP23 has a negligible contribution to overall signal intensity of the 3068 antibody in brain. Collectively, the results displayed in Figure 1 demonstrate the specificity of the 3067 and 3068 antibodies towards SNAP25a and SNAP25b respectively, and show that these antibodies reliably report on the relative expression levels of the endogenously expressed protein isoforms.

### Developmental expression of SNAP25a and SNAP25b in rat brain

**Figure 2:**
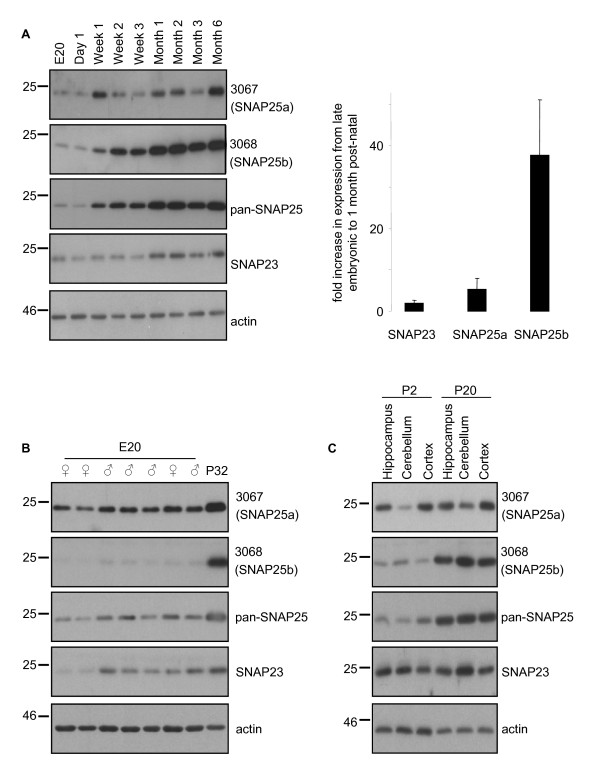
**Developmental expression patterns of SNAP25a and SNAP25b in rat brain**. A) *Left panel*, Equal amounts of whole brain lysates prepared from rats at different developmental stages (supplied by Zyagen Inc.) were resolved by SDS-PAGE and transferred to nitrocellulose for immunoblotting analysis using 3067, 3068, panSNAP25, SNAP23 and actin antibodies. *Right panel *shows the mean (+/-SEM) fold increase in expression levels of SNAP23, SNAP25a and SNAP25b between a late embryonic stage (E18-E20) and P30-32 (n = 3). The fold increase in SNAP25b expression was significantly greater than that of either SNAP25a (P < 0.05) or SNAP23 (p < 0.02), analysed using a one-way ANOVA. B) Equal amounts of brain lysates from gender-determined E20 litter mates were resolved on SDS-PAGE gels beside an aliquot of P32 brain lysate and analyzed as in panel A. *C*) Lysates from the hippocampus, cerebellum and cortex were prepared from pooled samples collected from 3 animals at P2 or P20. Samples were analyzed as in Panel A. Position of molecular weight standards are shown on the left of all panels.

SNAP25 displays a marked increase in expression from around post-natal week two in mice, and analysis of mRNA levels demonstrated a specific increase in the SNAP25b transcript at this stage [15]. However, expression of SNAP25a/b proteins will be affected by both transcriptional and post-transcriptional (e.g. protein stability) effects. Therefore, we examined how SNAP25a/b protein levels change during post-natal development. Brain lysates prepared from Sprague Dawley rats at different ages were probed with the 3067 and 3068 antibodies. SNAP25b exhibited a dramatic up-regulation in expression beginning at around post-natal week two (Figure 2A); similar increases in SNAP25b expression were observed in every set of samples that we tested. SNAP25a also exhibited a developmental increase but this was more modest and variable than SNAP25b (Figure 2A). Expression levels of SNAP23 were relatively constant throughout development (Figure 2A). For quantification purposes, we compared mean values of the fold increase in brain expression levels of the SNAP25/23 isoforms between a late embryonic stage (E18-E20) and ~ 1 month after birth (P30-P32). The graph in Figure 2A highlights the robust increase in SNAP25b levels during this period compared with SNAP25a and SNAP23. No gender-specific differences in SNAP25a/b expression levels were apparent at E20 (Figure 2B).

We next examined if the developmental increase in SNAP25b levels was similar in different brain regions. The hippocampus, cerebellum and cortex were collected from rats at P2 and P20 (3 animals per age) and lysed. Figure 2C reveals that SNAP25b expression increased between P2 and P20 to a similar extent in each brain region tested.

### Regional expression of SNAP25a and SNAP25b in rat brain

**Figure 3:**
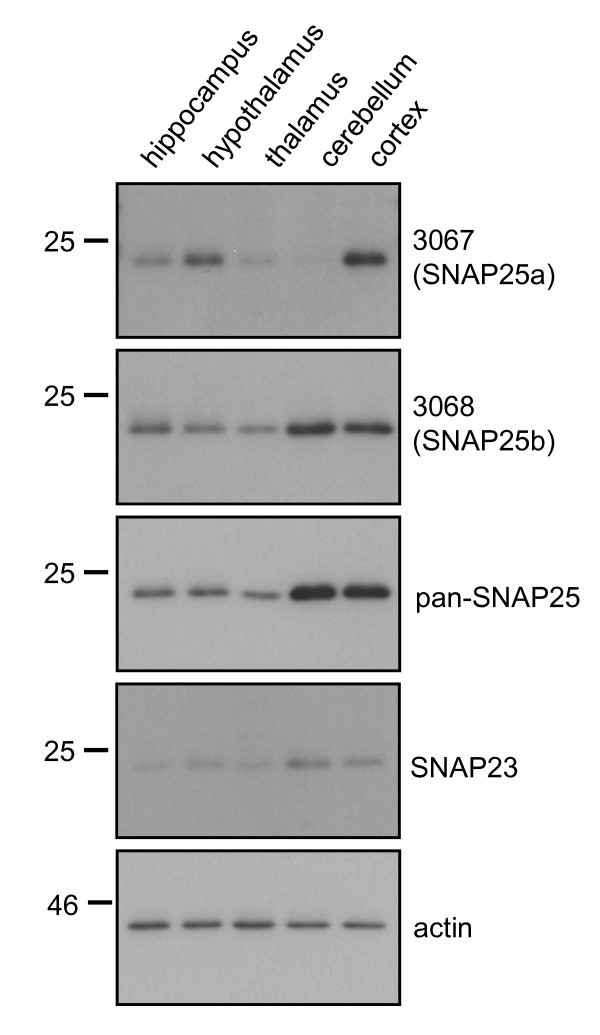
**Regional expression of SNAP25a and SNAP25b in rat brain**. Lysates from various brain regions (supplied by Zyagen; 3 animals/sample; ~ 10 week old) were resolved by SDS-PAGE and transferred to nitrocellulose for immunoblotting analysis using antibodies against SNAP25a (3067), SNAP25b (3068), panSNAP25, SNAP23 or actin. Position of molecular weight markers are shown on the left side of all blots.

To compare the regional expression levels of SNAP25a and SNAP25b in brain, samples of hippocampus, hypothalamus, thalamus, cerebellum and cortex from ~10 week-old rats were analyzed by immunoblotting. SNAP25a displayed highest levels (per μg of total protein) in cortex and hypothalamus (Figure 3), whereas SNAP25b had the highest immunoreactivity in cortex and cerebellum lysates. A marked difference in regional expression levels between the SNAP25 isoforms was detected in cerebellum: SNAP25b expression was highest in this brain region, whereas SNAP25a expression was lowest (Figure 3). Probing the brain region lysates with a panSNAP25 antibody revealed that total SNAP25 expression levels mirrored those of SNAP25b (Figure 3, *third panel*), consistent with the idea that SNAP25b is the major SNAP25 isoform expressed in adult brain.

### Regional expression and semi-quantitative comparison of SNAP25a, SNAP25b, and SNAP23 in human brain

**Figure 4:**
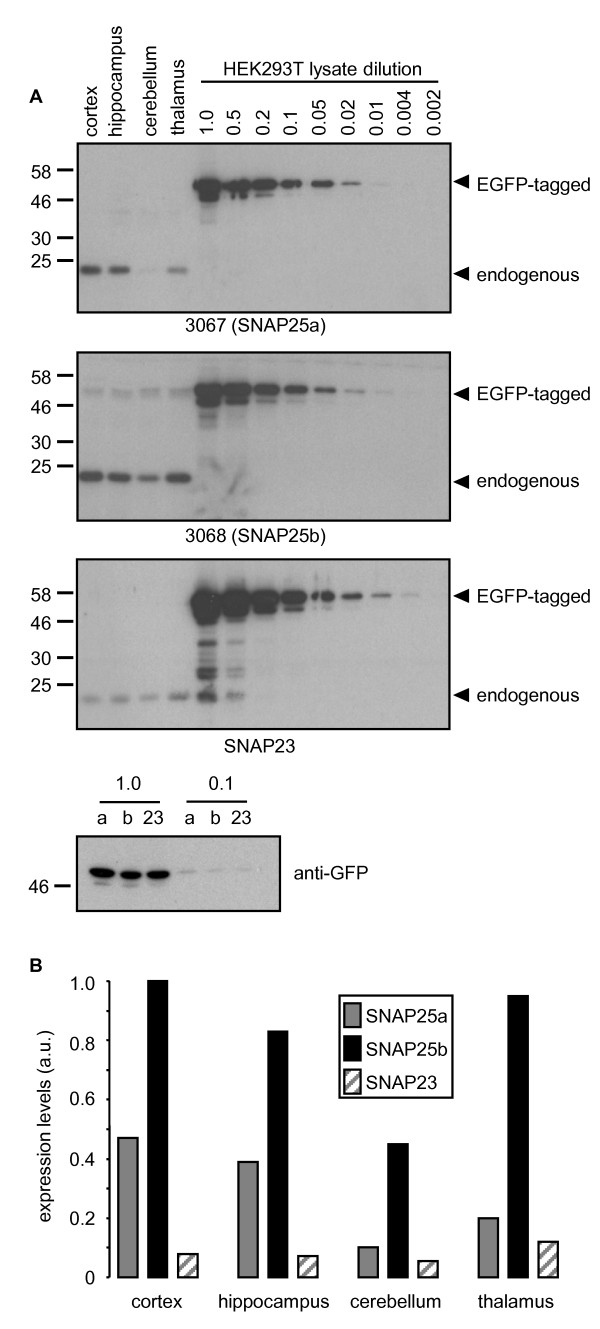
**Semi-quantitative comparison of SNAP25 isoform expression levels in human brain**. A) Lysates prepared from post-mortem brain samples (pooled from 6 individual patients) were resolved by SDS-PAGE alongside various dilutions of HEK293T lysates from cells transfected with EGFP-SNAP25a, EGFP-SNAP25b or EGFP-SNAP23. Gels were transferred to nitrocellulose and immunoblotted using antibodies against SNAP25a (3067), SNAP25b (3068) or SNAP23. The position of EGFP-tagged and endogenous proteins are highlighted, and molecular weight markers are shown on the left side of all blots. B) The relative levels of SNAP25a, SNAP25b and SNAP23 in brain were calculated by comparison of band intensities to those of the respective EGFP-tagged proteins. Highest expression levels were for SNAP25b in cortex and this was given an arbitrary value of 1, with all other values expressed as a fraction of this.

Having characterized regional expression patterns of SNAP25a and SNAP25b in rat brain, we next examined expression in post-mortem samples of human brain. For this, lysates of cortex, hippocampus, cerebellum and thalamus were prepared from 6 post-mortem brains. Equal amounts of the individual lysates from each brain region were then pooled and resolved on SDS-PAGE gels alongside a dilution series of HEK293T lysates from cells transfected with EGFP-SNAP25a, EGFP-SNAP25b or EGFP-SNAP23 (Figure 4A). Densitometry was then used to estimate the relative expression levels of SNAP25a, SNAP25b and SNAP23 in the different brain regions by comparison with the EGFP-tagged standards (Figure 4B). This semi-quantitative analysis revealed that expression of SNAP25b was highest and SNAP23 expression lowest in every brain region examined. The expression pattern of SNAP25a in human brain was grossly similar to that observed in rat brain, with this isoform highest in cortex and only weakly expressed in cerebellum.

## Discussion

The generation of antibodies that distinguish between SNAP25 splice variants is an important development that will significantly advance the study of these protein isoforms. RNA analysis provides a measure of relative protein expression levels but this can be complicated by differential protein turnover and stability. Antibodies also offer scope to study the intracellular localization of the endogenously expressed proteins, and to determine if the distinct cysteine-rich domains of SNAP25a/b mediate different intracellular sorting patterns; these analyses are ongoing in our laboratory. Cross-reactivity of the SNAP25b antibody (3068) with SNAP23 was unavoidable. However, Figure 1C and Figure 4 demonstrate that SNAP25b is expressed at much higher levels than SNAP23 in brain, and that the contribution of SNAP23 to signal intensity with the 3068 antibody is negligible. However, cross-reactivity is likely to be more of an issue when using the 3068 antibody on samples other than brain, or in brain areas where SNAP25b is weakly expressed.

Previous work had shown that SNAP25b mRNA is selectively up-regulated during post-natal development [15,24]. Our study is in broad agreement with these findings. However in some experiments we did also detect a marked increase in SNAP25a expression; we currently do not know the reason for this variability. Nevertheless, these observations suggest that mRNA analyses may not provide a complete picture of SNAP25a/b expression levels and indicates that the stability of SNAP25a protein may change during post-natal development. The increased post-natal expression of SNAP25b was similar in hippocampus, cerebellum and cortex, implying that changes in expression of this isoform are largely occurring at a global level.

SNAP25a and SNAP25b displayed differential patterns of expression in gross brain regions. In rat brain, a marked difference was found in cerebellum, where SNAP25b was highly expressed and SNAP25a was only present at low levels. The weak expression of SNAP25a in cerebellum was also observed in post-mortem samples from human brain. It is possible that SNAP25b is the more evolutionarily-ancient of the two SNAP25 isoforms given that SNAP25b shares higher sequence identity with SNAP23 (Figure 1A). These new antibodies offer scope to extend upon these observations, for example, by analyzing how the isoforms are distributed in more defined sub-regions of the brain or in specific neuronal populations.

The increased level of exocytosis supported by SNAP25b in mouse embryonic chromaffin cells was shown to be attributable to splicing differences that occur upstream of the palmitoylated cysteines [22,26,27]. These results might suggest that differential palmitoylation of SNAP25a and SNAP25b does not impact on the functionality of the isoforms. However functional outcomes attributable to differences within the palmitoylated cysteine-rich domains of SNAP25a/b will probably arise via changes in membrane interaction and/or lateral organization in the membrane [16,28,29]. These parameters are likely to be saturated by the high level over-expression (approximately 20-fold) achieved in these studies. Thus, there is some merit in studying the expression and localization of SNAP25a/b at high resolution. In particular, the use of specific antibodies against these proteins will be important to analyse patterning of the endogenous proteins and thus without any confounding effects of over-expression or fluorescent tags. Similarly, it will be interesting in the future to assess whether functional effects related to the different cysteine-rich domains of these SNAP25 isoforms are apparent when the proteins are expressed at endogenous levels.

At a similar time when this work was submitted for publication, a paper was published by the Takahashi group also describing the production of isoform-specific antibodies against the SNAP25 family and their use to analyse expression of the proteins in mouse brain [30]. Similar to our study, this study reported relatively low expression levels of SNAP25a in cerebellum, and that SNAP25b is the major isoform expressed in brain (~10-fold higher than SNAP25a in hippocampus).

## Conclusions

We have shown that SNAP25a and SNAP25b proteins display distinct regional and developmental profiles in rat and human brain. This analysis will be helpful in further delineating how the function of these isoforms overlaps and/or is distinct. The antibodies generated in the study will serve as useful tools for future work in this area.

## Methods

### Chemicals

Lipofectamine 2000 was purchased from Invitrogen (Paisley, UK). COMPLETE Protease inhibitor cocktail tablets were from Roche (Buckinghamshire, UK). NP40, acrylamide and all other reagents were of an analytical grade from Sigma (Poole, UK).

### Antibodies

Peptide synthesis and antibody production was performed by Eurogentec; the sequences of peptides used for immunization are shown in Figure 1A. IgG fractions from recovered antisera were purified on protein A agarose and dialyzed against phosphate buffered saline (PBS). Purified IgGs were aliquoted and stored at -80°C. For immunoblotting, the purified IgGs were used at a final concentration of 1 μg/ml in PBS supplemented with 0.05% Tween-20. Antibody incubations were performed as standard for 60 min at room temperature with gentle shaking.

Rabbit SNAP23 antibody and mouse monoclonal SNAP25 antibody were from Synaptic Systems (Gottingen, Germany). Monoclonal actin antibody was purchased from Abcam (Cambridge, UK). Monoclonal anti-GFP (JL8) was from Roche (Buckinghamshire, UK).

### Plasmids

Plasmids encoding SNAP25a, SNAP25b and SNAP23 tagged N-terminally with EGFP have been previously described [14].

### Cell culture and transfection

HEK293T cells were obtained from ATCC and cultured in DMEM with 10% fetal bovine serum in a humidified atmosphere containing 5% CO_2_. Cells were transfected using Lipofectamine 2000 (Invitrogen, Paisley, UK) according to the manufacturer's instructions.

### Animals

Unless otherwise stated, all samples used were collected from in-house bred Sprague Dawley rats, following internationally recognise guidelines. The rat brain region lysates that were analyzed in Figure 3 were obtained from Zyagen (San Diego, USA). These samples represent pooled fractions from at least 3 different Sprague Dawley rats, approximate age 10 weeks old. Zyagen also supplied the developmental samples that were analyzed in Figure 2A. These samples are whole brain lysates from one Sprague Dawley rat of each age. The cerebellum samples from wild-type and SNAP25^a/a ^mice were kindly supplied by Dr Christina Bark (Karolinska Institute, Sweden).

### Preparation of tissue lysates and SDS-PAGE

Brain lysates were prepared by homogenization of samples in ice-cold buffer A [20 mM HEPES, 1 mM MgCl_2_, 250 mM sucrose, 2 mM EDTA, 1% NP-40 and protease inhibitor cocktail (Roche), pH 7.4] using a Dounce homogenizer. Insoluble material was removed by centrifugation 4,000 xg for 2 min. The protein content of recovered lysates was determined, and equal amounts of the various lysates were resolved by SDS-PAGE and transferred to nitrocellulose for immunoblotting analysis.

#### Human post-mortem brain samples

Frozen samples from histologically characterised normal brain tissues authorised for ethically approved scientific research (Lothian Research Ethical Committee; ref. 2003/8/37) were gratefully provided by Robert Walker at the Medical Research Council Sudden Death Brain and Tissue Bank, University of Edinburgh [26], and studied in collaboration with Douglas Blackwood and Donald McIntyre, Division of Psychiatry, University of Edinburgh (ethical approval reference MREC/99/0/12). Tissue samples from 6 post-mortem brains were dissected from the cortex, hippocampus, cerebellum and thalamus. Lysates were prepared as described for the rat brain samples.

## Abbreviations

SNAP25: synaptosomal-associated protein of 25 kDa; SNAP23: SNAP25 homologue of 23 kDa.

## Competing interests

The authors declare that they have no competing interests.

## Authors' contributions

Both authors read and approved the manuscript. LC conceived of the study, designed peptides for antibody production, performed an initial characterisation of the antibodies and wrote the manuscript. GP contributed to the study design, performed the experiments and analysed the data, and helped draft the manuscript.
